# Misdiagnosis of soft tissue sarcomas of the lower limb associated with deep venous thrombosis: report of two cases and review of the literature

**DOI:** 10.1186/1471-2474-14-64

**Published:** 2013-02-19

**Authors:** Carlo Perisano, Nicola Maffulli, Pamela Colelli, Emanuele Marzetti, Alfredo Schiavone Panni, Giulio Maccauro

**Affiliations:** 1 Department of Geriatrics, Neurosciences and Orthopedics, University Hospital “Agostino Gemelli”, Catholic University of the Sacred Heart School of Medicine, L.go A. Gemelli 1, 00168, Rome, Italy; 2 Centre for Sports and Exercise Medicine, Queen Mary, University of London Barts and The London School of Medicine and Dentistry, Mile End Hospital, 275 Bancroft Rd, London, E1 4DG, UK; 3 Department of Radiology, Hospital ”A.Perrino”, S.S.7 – 72100, Brindisi, Italy; 4 Department of Health Science, University of Molise, Campobasso, Italy

**Keywords:** Leiomyosarcoma, Venous thromboembolism, Lower extremity sarcoma, Pulmonary embolism

## Abstract

**Background:**

Deep venous thrombosis (DVT) or pulmonary embolism (PE) is a rare, but not exceptional presentation of soft tissue sarcomas (STSs). Due to the remarkable difference in the incidence between DVT or PE and STSs, this type of STS presentation is usually associated with a considerable delay in tumor diagnosis and treatment.

**Case Presentation:**

We describe two cases of STS who presented with DVT and PE. Physical and radiographic examination only showed the presence of DVT. Both patients were treated for DVT or PE for several months. Due to the persistence of symptoms and the inefficacy of anticoagulant therapy, magnetic resonance imaging (MRI) was performed, which revealed the presence of a lower limb mass in both cases. The definite diagnosis was reached via excisional biopsy and histological examination.

In one case, MRI showed a large tumor in the anterior muscle compartment of the right thigh, with thrombosis of the right common femoral vein and involvement of the ipsilateral common iliac vein and inferior vena cava until the confluence of the renal veins. In the other case, MRI showed a large tumor in the middle third of the right thigh. The lesion was in close proximity to the superficial femoral vein that appeared compressed and showed signs of thrombosis. In both cases, histological examination revealed a high-grade leiomyosarcoma.

**Conclusion:**

STSs of the lower extremities can rarely present with DVT or PE. This possibility should be considered in the differential diagnosis of painful leg swelling, especially in patients with recurrent or refractory venous thrombosis. When a STS is suspected, MRI should be obtained followed by excisional biopsy of the eventual mass. A delay in diagnosis and treatment of STSs often results in very poor prognosis.

Level of evidence. IV

## Background

Sarcomas are relatively rare malignant tumors arising from the mesenchymal tissue, which encompasses muscle, fat, bone, blood vessels, and fibrous or other supporting tissue. Sarcomas display a wide variety of histological subtypes and frequently involve the limbs (55% of cases), especially the lower extremities [1-3].

The incidence of soft tissue sarcomas (STSs) is approximately 15-35 cases per million person-years, accounting for less than 1% of all malignancies [1-3]. About 3,200 new cases of STSs are diagnosed yearly in the U.K., while approximately 10,600 new cases were discovered in the U.S. in 2009 [4,5]. The incidence of STSs increases steadily with age and is slightly higher in males than females. However, marked differences in age and sex distribution are seen depending on the histotype. For instance, rhabdomyosarcoma is more frequently observed in children and young adults, whereas synovial sarcomas typically affect young adult people. Malignant fibrous histiocytoma and liposarcoma generally occur in older adults [4,5].

Overall, the risk of thromboembolic events in patients with STSs is comparable with that observed in other orthopedic conditions. However, STSs involving the hip or the thigh have been associated with a particularly high risk of thromboembolism [6].

Deep venous thrombosis (DVT) and venous thromboembolism (VTE) are common causes of morbidity and mortality especially in bedridden or hospitalized patients. In the U.S., the incidence of DVT is approximately 80-100 cases per 100,000 person-years [7]. The risk of DVT increases with advancing age, ranging from less than 5 cases per 100,000 person-years in kids younger than 15 years to more than 500 cases per 100,000 person-years in 80+ year-old persons [7,8]. DVT is responsible for about 600,000 hospitalizations yearly in the U.S. The in-hospital case-fatality rate for VTE is 12% and rises up to 21% in elderly individuals. The incidence of DVT is considerably higher in hospitalized patients compared with community-dwellings and varies from 20-70% [7,8]. The risk of thromboembolic events is much greater in cancer patients relative to the general population [9,10]. Indeed, patients with cancer are at 4- to 7-fold higher risk for VTE than those without cancer, with about 15% of cancer patients suffering at least a VTE episode. Notably, approximately 20% of patients presenting with VTE have an active cancer [11].

DVT is a rare, but not exceptional presentation of a STS. Due to the remarkable difference in the incidence of the two conditions, this type of presentation is usually associated with a considerable delay in the diagnosis and treatment of the tumor. In the present study, we describe 2 cases of STS of the lower limb, the onset of which was characterized by DVT and VTE. Because of the unusual presentation, in both cases diagnosis and treatment of the STS were significantly delayed. We also provide a concise review of the literature about the association between STSs and VTE.

Ethical approval was obtained by Ethical Committee of the Catholic University of Sacred Heart in Rome.

## Case presentation

**Figure 1:**
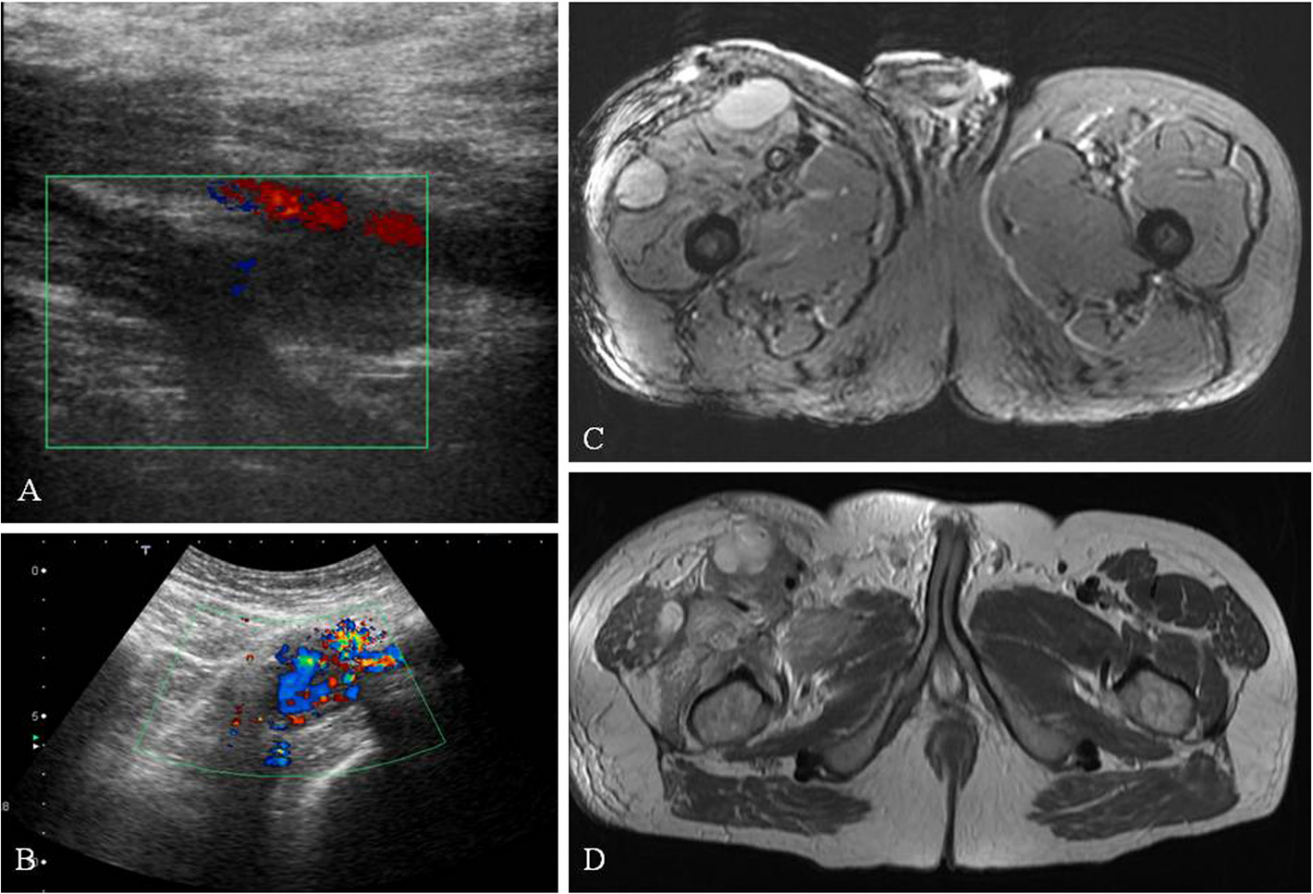
**A,B: Doppler ultrasonography showing thrombosis of the right femoral and iliac vein with artero-venous microfistulas.C,D:** MRI of the femur showing a leiomyosarcoma with thrombosis of the superficial and common femoral veins and involvement of the internal iliac vein.

**Figure 2:**
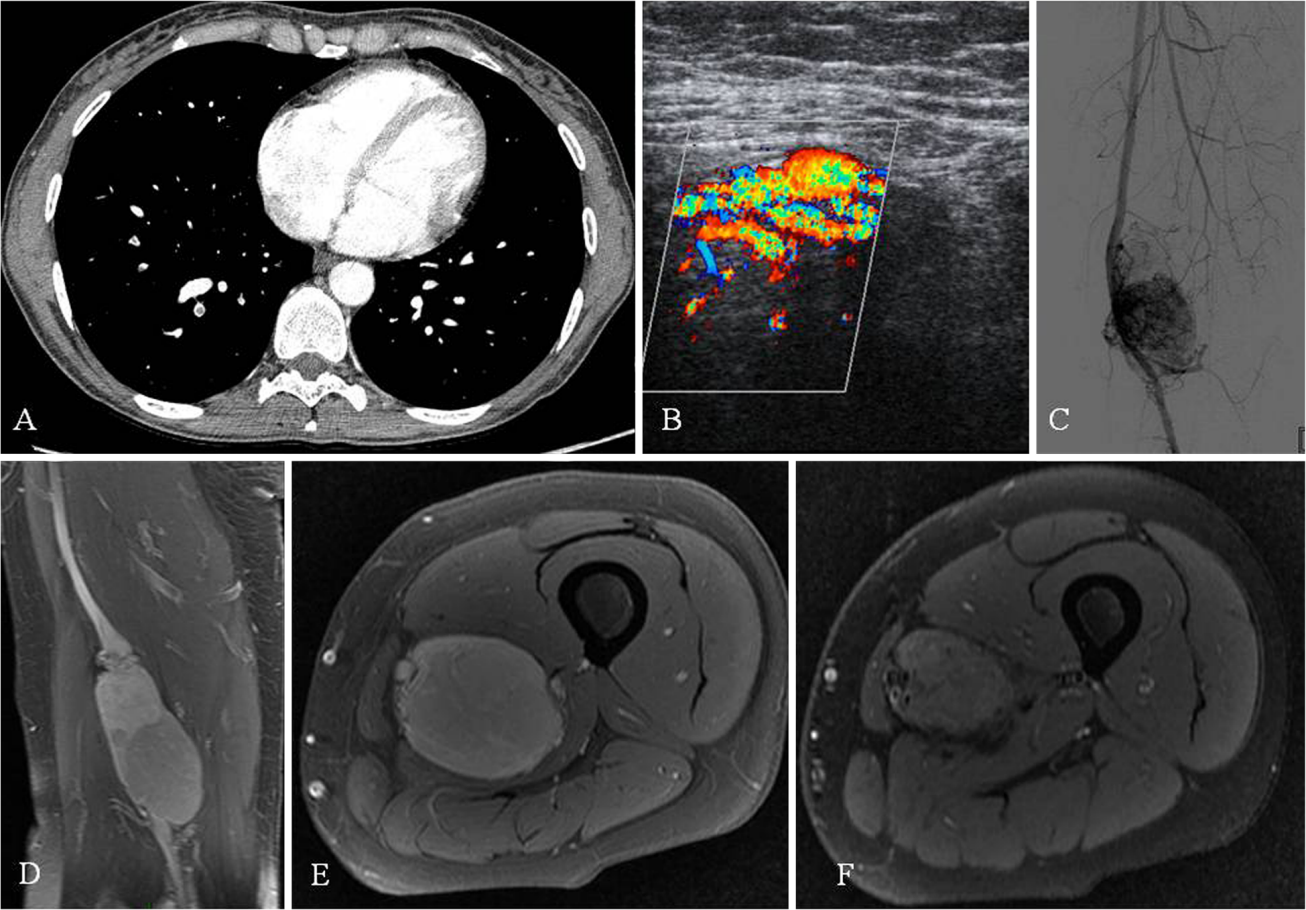
**A: CT scan showing PE. B:** Doppler ultrasonography showing hypervascularization of the lesion. **C:** Arteriography showing the location of the lesion adjacent to the superficial femoral artery and the superficial femoral vein. **D,E,F:** MRI of the femur showing a mass close to the superficial femoral artery and the superficial femoral vein that appears compressed and deformed.

Two cases of STS of the lower limb characterized by DVT and pulmonary embolism (PE) as the initial presentation were observed in our Orthopedic department: a 47-year-old man diagnosed with an idiopathic DVT of the right distal femoral vein and the popliteal vein (Figure 1A-B) and a 44-years-old woman with a massive PE (Figure 2A). The patient with DVT was treated with therapeutic doses of subcutaneous enoxaparin for 6 months. After 2 months of enoxaparin, the case with PE was referred to our outpatient clinic for worsening of leg swelling and dyspnea. Neither of the patients had a past or family history of VTE. Physical and radiographic examination did not reveal any mass in either case, but only the signs of DVT or PE. Due to the persistence of symptoms and the inefficacy of anticoagulant therapy, patients underwent magnetic resonance imaging (MRI) of the lower limbs and excisional biopsy of the visualized mass.

In the first case (Figure 1C-D), MRI showed a large mass in the anterior muscle compartment of the right thigh, with inhomogeneous appearance after gadolinium administration. The lesion was associated with multiple lymphadenopathy in the inguinal and external iliac region, and thrombosis of the right common femoral vein involving the ipsilateral common iliac vein and the inferior vena cava until the confluence of the renal veins. An excisional biopsy of the mass was performed. The lesion was found to be adherent to the femoral vein. The dissection of the vein showed a thrombus that obliterated the lumen of the common iliac vein. The histological examination revealed a high-grade leiomyosarcoma. The patient subsequently underwent chemotherapy and radiotherapy. A computerized tomography (CT) scan performed 6 months after surgery showed multiple pulmonary metastases. The patients is currently receiving chemotherapy in the Oncology division of our hospital.

In the second case, MRI showed a large mass located in the middle third of the right thigh, with low signal intensity in T1- and T2-weighted sequences and enhancement after gadolinium administration (Figure 2B-C-D-E-F). The lesion was in close proximity to the superficial femoral artery that appeared deformed and was adherent to the superficial femoral vein that appeared compressed and showed signs of thrombosis. After arterial embolization, an excisional biopsy was performed, followed by intraoperative brachytherapy. The histological examination of the bioptic specimen revealed a high-grade leiomyosarcoma. At one year of follow-up the patient was asymptomatic and showed no evidence of recurrence of malignancy at MRI.

## Literature review

A PubMed search was performed using the terms “soft tissue sarcoma”, “deep venous thrombosis”, and “lower limb sarcoma”. The search retrieved three case series and several case reports describing STSs characterized by DVT as the initial presentation (Table 1) [12-29]. No reports have been published that describe STSs presenting with PE.

**Table 1:** Publications indexed in PubMed on STSs of the lower extremities associated with DVT, ordered according to the number of cases described

**Publication**	**Histotype**	**Total number of cases**	**Reference number**
Benns M et al. J Vasc Surg. ([2006])	Malignant fibrous histiocytoma (3 cases)	6	14
	Leiomyosarcoma (1 case)		
	Pleomorphic sarcoma (1) (1 case)		
	Angiosarcoma (1)		
Arumilli et al. World J Surg [Oncol (2008])	Pleomorphic sarcoma (1 case)	3	15
	Giant cell tumor ( 1 case)		
	Liposarcoma (1 case)		
Emori M et al. Ann Vasc Surg. ([2010])	Malignant fibrous histiocytoma (1 case)	2	16
	Synovial sarcoma (1 case)		
da Gama AD et al. Rev Port Cir Card [Vasc (2009])	Leiomyosarcoma	1	17
Durant C et al. J Mal [Vasc (2009])	Leiomyosarcoma	1	18
Subramaniam MM et al. Virchows Arch. ([2007])	Leiomyosarcoma	1	19
Sakpal SV et al. J Vasc Surg. ([2009])	Leiomyosarcoma	1	20
Calderelli GF et al. J Vasc Surg. ([2003])	Leiomyosarcoma	1	21
Shindo S et al. J Vasc Interv Radiol. ([2009])	Leiomyosarcoma	1	22
Tasci I et al. Clin Transl [Onc (2008])	Ewing sarcoma	1	23
Greenwald U et al. Ann Vasc [Surg (2004])	Angiosarcoma	1	24
Carter RM et al. Clin [Radiol (2008])	Spindle cell sarcoma	1	25
Hoekstra HJ et al. Eur J Surg [Oncol (1997])	Chondrosarcoma	1	27
Minami S et al. J Orthop Sci. ([2011])	Chondrosarcoma	1	28
Singh NK et al. [Phlebology (2009])	Liposarcoma	1	29

In a large case series of 5,234 patients treated for STSs, 19 cases exhibited the coexistence of STS and DVT (prevalence: 0.36%) [14]. Among these patients, 6 cases (0.11%) were diagnosed with DVT before the STS had been discovered. In 3 cases, the histotype was malignant fibrous histiocytoma, whilst leiomyosarcoma, pleomorphic sarcoma and high-grade angiosarcoma were diagnosed in the remaining 3 cases [14]. In 5 of these cases there was a delay in STS diagnosis ranging from one day to 12 months [14].

Arumilli et al. [15] describe 3 cases who presented with a painful swollen leg and were initially treated for DVT or Baker’s cyst, but were later diagnosed with a pleomorphic sarcoma, a malignant giant cell tumor of the muscle, and a myxoid liposarcoma. In another small case series, two cases of STSs of the inguinal region are described with compression of femoral vessels and venous obstruction [16]. The condition resulted in signs and symptoms mimicking a DVT, including swelling, pain, and skin discoloration of the affected limb. Both patients were initially diagnosed with spontaneous DVT and administered unnecessary long-term anticoagulant therapy.

Other reports describe cases of leiomyosarcoma of the femoral vein, leiomyosarcoma involving the profunda femoris artery and the popliteal artery, fibular Ewing’s sarcoma, malignant epitheliod angiosarcoma, spindle cell sarcoma, pelvic and sacrum condrosarcoma, and liposarcoma [17-29]. In all these cases, patients presented with DVT of the lower extremity with initial misdiagnosis, delayed treatment, and poor outcome.

## Discussion

Both the presence of cancer and cancer treatment, including surgery and chemotherapy, are major risk factors for DVT. Indeed, approximately 18% of incident DVT cases can be attributed to an active malignant neoplasm [30]. Remarkably, about 15% of pediatric or young adult patients with bone sarcomas or STSs develop thromboembolic events [31,32]. Sarcomas located in the hip or the thigh are associated with a particularly high risk of thromboembolism [6]. These observations indicate that DVT is a common event in patients affected by sarcomas of the lower extremities. In such circumstances, DVT may occur as a result of venous compression by the mass or due to a hypercoagulable state secondary to the production and secretion of procoagulant/fibrinolytic substances and inflammatory cytokines by cancer cells [33].

However, DVT is an unusual presentation STSs [23]. The differential diagnosis between a thrombus and a STS by CT and MRI may be a challenging task, because imaging cannot easily distinguish a tumor thrombus from a blood clot. Furthermore, an organized thrombus usually shows low signal intensity on T1-and T2-weighted MRI sequences [22], which may be similar to the pattern evoked by a STS.

The two cases of leiomyosarcoma observed in our department support the notion that this histotype is the most frequently associated with unexplained DVT. The excision of the tumor followed by chemotherapy/radiotherapy or brachitherapy led to complete resolution of DVT and PE.

In conclusion, STSs are rare, yet potentially fatal malignancies characterized by local extension and occasional distant metastases. STSs of the lower extremities can present with VTE or mimic DVT, which may result in substantial delays in the diagnosis and treatment of the tumor. The two cases described here, together with our concise literature review, highlight the importance of considering the presence of neoplastic masses in the differential diagnosis of painful leg swelling conditions. In particular, STSs should be excluded in young patients with no risk factors for VTE and in those with recurrent or refractory thrombosis. In these cases, a careful physical examination together with imaging-assisted techniques (e.g., ultrasonography and MRI) are of utmost importance to promptly identify an eventual STS. Such an approach ensures the early diagnosis and management of STSs, which is essential to minimize morbidity and mortality [34-36].

## Abbreviations

CT: Computerized tomography; DVT: Deep venous thrombosis; MRI: Magnetic resonance imaging; PE: Pulmonary embolism; STS: Soft tissue sarcoma; VTE: Venous thromboembolism.

## Competing interests

The authors declare that they have no conflict of interest.

## Authors’ contributions

All authors read and approved the final manuscript. All authors contributed equally to this work.

## Pre-publication history

The pre-publication history for this paper can be accessed here:

http://www.biomedcentral.com/1471-2474/14/64/prepub
